# Syndrome de Waardenburg

**DOI:** 10.11604/pamj.2015.20.427.6209

**Published:** 2015-04-29

**Authors:** Mahfoudhi Madiha, Khamassi Khaled

**Affiliations:** 1 Service de Médecine Interne A, Hôpital Charles Nicolle, Tunis, Tunisie; 2 Service ORL, Hôpital Charles Nicolle, Tunis, Tunisie

**Keywords:** Syndrome de Waardenburg, surdité de perception, dépigmentation, Waardenburg syndrome, sensorineural hearing loss, depigmentation

## Image en medicine

Le syndrome de Waardenburg associe une surdité à des anomalies de pigmentation. Ce syndrome est autosomique dominant à pénétrance et expressivité variable en inter et en intra familial, d'où l'intérêt du diagnostic prénatal dans les cas à risque. Le type I présente une association clinique comprenant au moins 2 critères majeurs ou au moins un critère majeur et 2 critères mineurs. La surdité neurosensorielle fait partie des critères majeurs. Le diagnostic biologique repose sur la recherche de mutation du gène PAX3 sur le chromosome 2q37. L'appareillage de la surdité et la prise en charge des malformations associées sont nécessaires. Patiente âgée de 3 ans, sans antécédents pathologiques notables, ni contexte familial de surdité, qui a consulté pour un retard de langage avec suspicion d'hypoacousie par la mère. A l'examen, elle avait une mèche de cheveux blanche, un hypertélorisme avec diminution de la longueur de la fente palpébrale, et des plaques dépigmentées au niveau de l'abdomen et des membres inférieurs. Les tympans étaient complets et normaux. L'examen ophtalmologique était sans anomalie. L'impédancemétrie était normale. Les potentiels évoqués auditifs ont révélé une surdité de perception bilatérale à 70 dB. Le diagnostic de syndrome de Waardenburg a été retenu devant l'association de plus de 2 critères majeurs (surdité de perception, dystopie des canthi, anomalies de dépigmentation). La patiente a bénéficié d'un appareillage auditif avec rééducation orthophonique.

**Figure 1:**
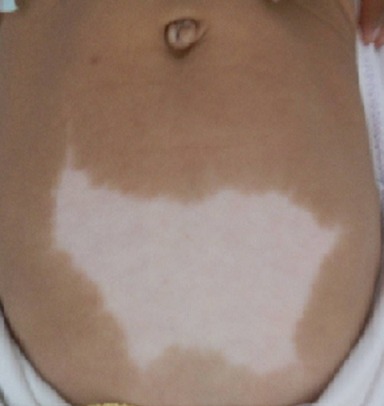
Plaque dépigmentée de l'abdomen

